# The “hyperdense basivertebral vein” sign: another marker of a CSF-venous fistula

**DOI:** 10.1007/s00234-022-02908-x

**Published:** 2022-02-01

**Authors:** Niklas Lützen, Nico Kremers, Christian Fung, Jürgen Beck, Horst Urbach

**Affiliations:** 1 Dept. of Neuroradiology, University Medical Center Freiburg, Faculty of Medicine, University of Freiburg, Breisacher Str. 64, 79106 Freiburg, Germany; 2 Dept. of Neurosurgery, University Medical Center Freiburg, Faculty of Medicine, University of Freiburg, Freiburg, Germany

**Keywords:** Cerebrospinal fluid, Spontaneous intracranial hypotension, CSF leak, CSF-venous fistulas, Myelography

## Abstract

CSF-venous fistulas — initially described in 2014 — are a significant cause of spontaneous intracranial hypotension. Dynamic lateral decubitus digital subtraction and/or CT myelography typically show a hyperdense paraspinal vein. In case of a central drainage toward the internal vertebral venous plexus, it is the “hyperdense basivertebral vein” which should be searched for carefully.

**Supplementary Information:**

The online version contains supplementary material available at 10.1007/s00234-022-02908-x.

## Introduction

Besides ventral dural tears and leaking meningeal diverticulas, CSF-venous fistulas (CVFs) — initially described by Schievink et al. in 2014 [1] — are a significant cause of spontaneous intracranial hypotension (SIH). A CVF is a direct connection of CSF to the vertebral venous plexus at the level of a nerve root sleeve. The most likely underlying cause is the rupture of an arachnoid granulation with a direct connection of CSF into an adjacent vein [2, 3].

Kranz et al. described the so called “Hyperdense Paraspinal Vein” sign on cross-sectional images in 2016 [5]: A hyperattenuated vein with density values of 60-140HU compared to a normal-appearing paraspinal vein at other levels indicating an underlying CVF. On CT myelography (CTM), abnormal veins of a CVF were seen in a paravertebral location in 45%, centrally within the internal vertebral venous plexus in 32%, and lateral to the spine in 23% of patients, respectively [2].

While the hyperdense paravertebral vein is a CT marker for a paravertebral and/or lateral CVF, it may be difficult to detect CVF draining centrally into the internal vertebral venous plexus [2, 3]. Here, we describe the “hyperdense basivertebral vein” as a CT marker of a central drainage of a CVF that might otherwise be easily overlooked.

Within an 8-month period, eight SIH patients undergoing a standardized diagnostic work-up [4] were diagnosed with a CVF. Two of them had a central drainage:

## Cases

### Case 1

**Fig. 1:**
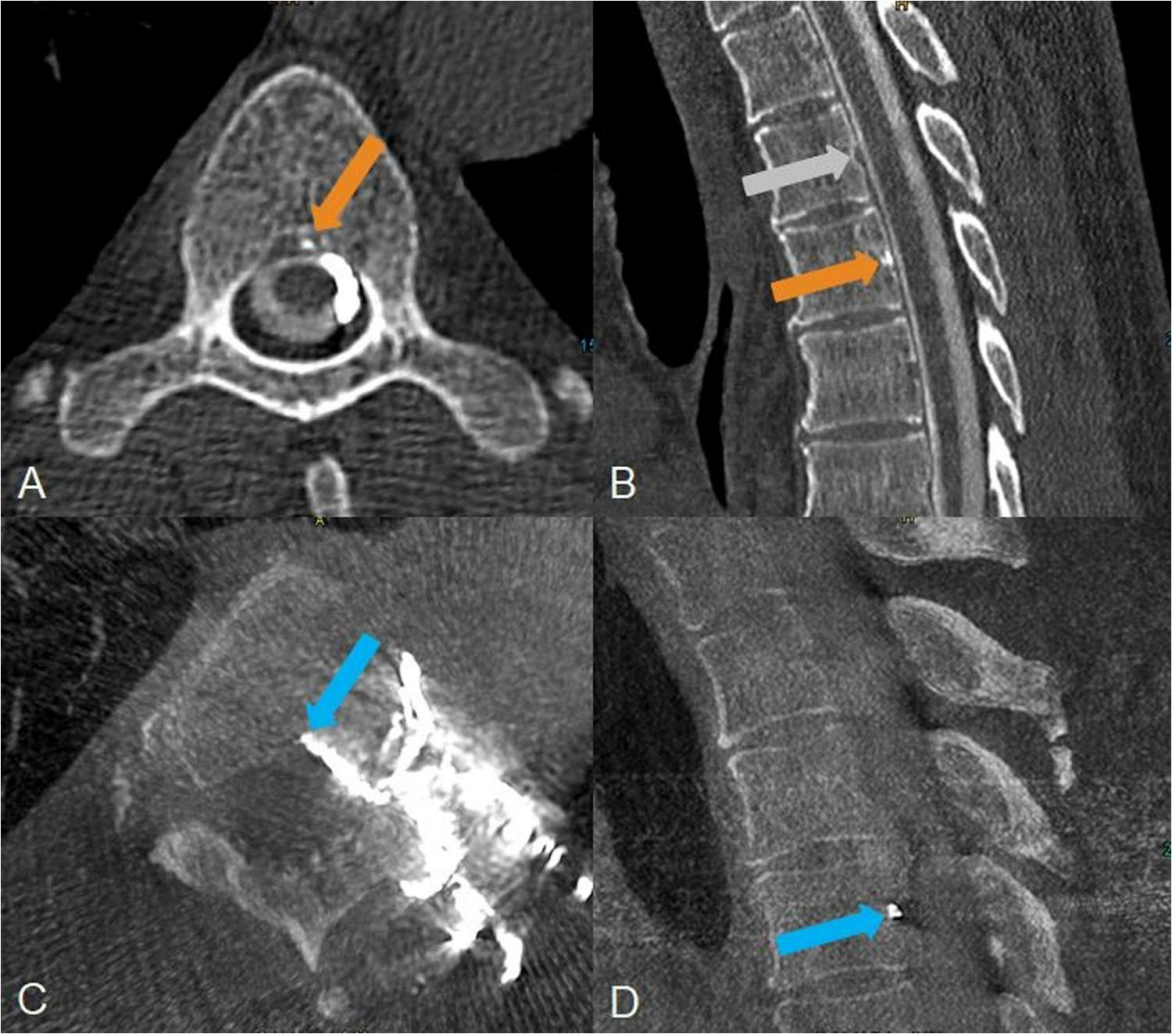
A 60-year-old man with a central CVF at the level of Th5/6 left. A, B Left lateral decubitus CTM shows a hyperdense spot in the axial and sagittal reformatted images (orange arrow) at the level of Th 5/6 representing the proximal “hyperdense basivertebral vein” sign. B, One level above e.g. the triangular shape of the basivertebral vein is empty (grey arrow). C, D After transvenous embolization the Onyx cast is confirmed to be within the central draining CVF in axial and sagittal reformatted images of a cone beam CT in which it fills the proximal basivertebral vein (blue arrow) brightly

A 60-year-old man presented with a sudden onset of increasing dizziness starting 17 days ago. He complained, as well, of fatigue and hearing loss as of several months. MRI of the brain was positive for SIH (“head positive”) meeting all signs of the so-called “Bern score” [6]. MRI of the spine did not reveal any spinal longitudinal extradural CSF collection (“SLEC negative”). Left-sided lateral decubitus CTM depicted a patchy hyperdensity with values of 1800 HU in the triangular-shaped proximal basivertebral vein and slightly in the adjacent left-sided anterior epidural venous plexus at the level of Th5/6 (Fig. 1). Intrathecal contrast column showed maximum 3100 HU (contrast medium concentration of 300 mg iodine/ml), maximum density of the surrounding bone was 1300 HU. Right-sided lateral decubitus CTM on the next day showed the basivertebral vein without contrast filling. Digital subtraction myelography (DSM) in the left lateral decubitus position did not reveal the CVF. After transvenous embolization of the CVF with Onyx, patient reported significant improvements of his symptoms whereas MRI of the head, however, continued to show SIH signs for which three causes are possible: the CVF was incompletely closed, another CVF has opened due to altered pressure conditions, or a new CVF has developed in the meantime [7].

### Case 2

**Fig. 2:**
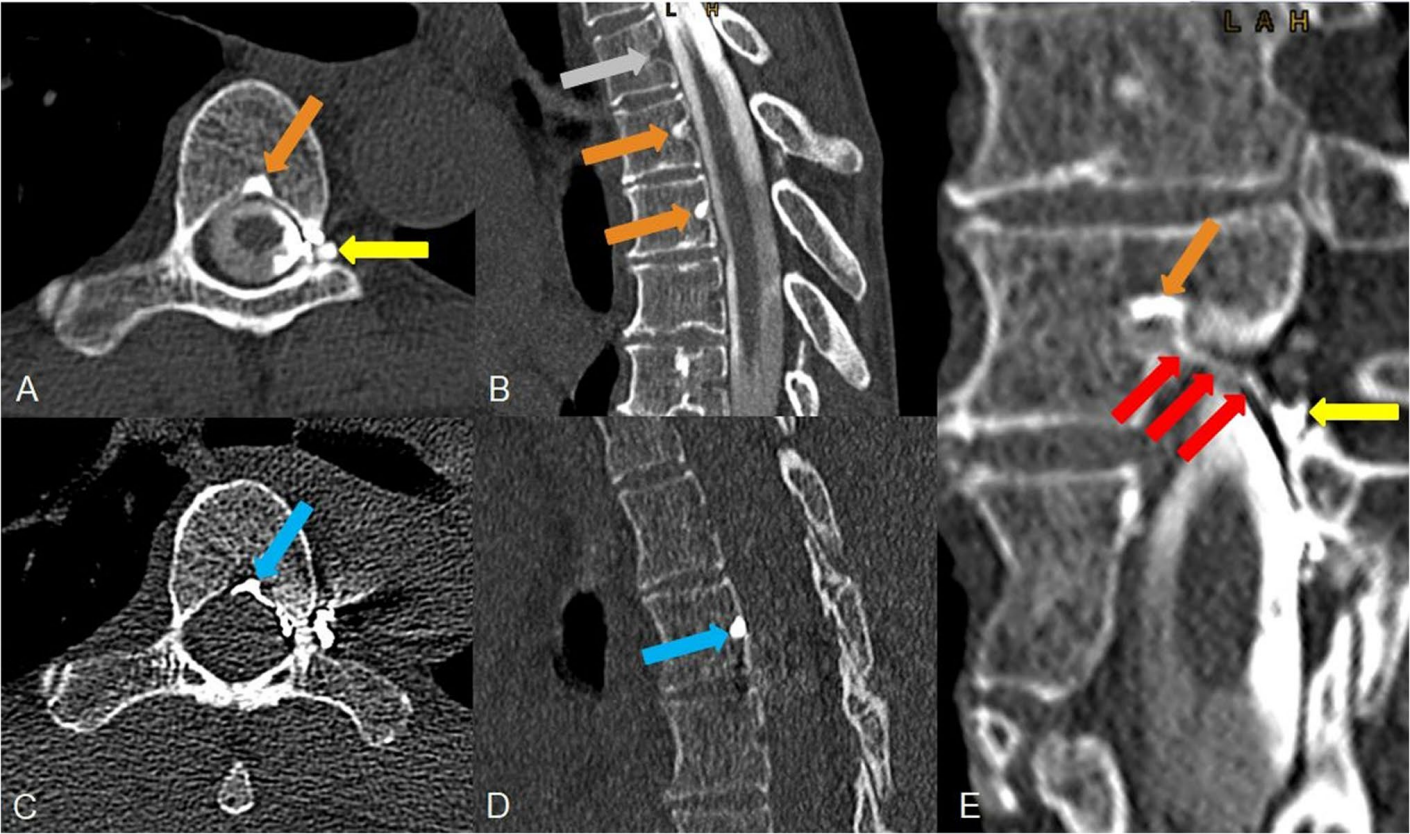
A 55-year-old woman with a central CVF at the level of Th5/6 left. A, B, Left-sided lateral decubitus CTM shows a “hyperdense basivertebaral ein” sign on the level of the CVF and one level above (orange arrows). An associated meningeal diverticulum is located at the left nerve root sleeve (yellow arrow). C, D Post endovascular embolization the Onyx cast can be confirmed in the basivertebral vein in question at the level of Th 5/6 (blue arrow). E, Curved planar reconstruction of the CVF on the CT scan reveals the central drainage from a spinal meningeal diverticulum on the left side (yellow arrow) representing the point of fistula, via the ventral epidural venous plexus at the left side (red arrows) to the proximal basivertebral vein (orange arrow)

A 55-year-old woman reported on sudden onset of strong orthostatic headache, nausea, vomiting, tinnitus and ear pressure starting 5 months ago. MRI scan was “head positive” (Bern score of 3), spine MRI was SLEC negative. Lateral decubitus CTM disclosed a hyperdense basivertebral vein at the level Th5/6 (Fig. 2), via an accessory paravertebral connection, the basivertebral vein on the level above filled as well. This accessory paravertebral vein of the CVF was present in the DSM as well (not shown), the central drainage, on the other hand, was not seen using this modality. Opacity of the contrast medium in the basivertebral vein was maximum 2600 HU, at the side of the intrathecal contrast column maximum 3100 HU, a lot denser than bony structures or calcifications. Complementary right-sided decubitus CTM the next day confirmed an “empty basivertebral vein” at these levels. After the patient had undergone transvenous occlusion symptoms completely resolved but rebound-hypertension occurred. MRI 6 weeks after embolization showed a declining SIH score from 3 to 1.

## Discussion

The relative incidence of CVFs has increased due to improved diagnostic work-up using lateral decubitus DSM and/or CTM [2, 4, 5], (see Table 1 in Supplementary appendix for DSM and CTM protocol details at our institution). Currently, CVFs are thought to be present in up to 25% of all causes of SIH [8]. Nevertheless, the detection of CVFs is still very challenging but crucial to provide effective therapy. The latest therapeutic approach is promising: a transvenous embolization with Onyx [9].

The hyperdense paraspinal vein is the classic marker of a CVF on cross-sectional images.

It occurs when intrathecal contrast fills the external vertebral venous plexus, which on CT is reasonably clear when present and contrast is high (see Fig. 3 in the Supplementary appendix for a typical CT image of a paravertebral CVF). Kranz et al. (2) described 3 types of CVFs (paravertebral, lateral or central drainage) which definitely can occur in combination. In case of a central CVF, a paraspinal vein can be normal while the epidural venous plexus and/or basivertebral vein becomes hyperdense. Whether or not this basivertebral vein is hyperdense in a central CVF likely depends on the timing of CT acquisition. Accordingly, this report describes one aspect of CT diagnosis of a central draining CVF, which in our opinion is the most difficult type of CVFs to detect on CT and sometimes requires meticulous searching.

The basivertebral vein is located centrally within the vertebral body and carries blood from the internal (epidural) vertebral venous plexus to the external vertebral venous plexus. In sagittal reformatted images, the proximal basivertebral vein at midline posterior surface of the vertebral body has a triangular shape in which contrast material can pool if a central CVF is present. Due to the anatomical conditions, a central drainage of a CVF, starting from the fistula point at the nerve root sleeve, may be easily missed even by experienced neuroradiologists: The epidural space is narrow, the hyper attenuated vein is small and short (see Fig. 2E) and could be misinterpreted as intraspinal calcification or bony spur. Therefore, the central draining CVFs might be underdiagnosed. The proximal hyperdense basivertebral vein as described in this report is a marker to detect central CVFs and may be the only indication of its presence.

Midsagittal CT reconstructions could easily point out these clues at a glance as one can compare the proximal triangular part of the basivertebral veins on different levels (Figs. 1, 2). We propose to implement midsagittal views in a structured review of CT images to increase the detection rate of CVFs. Of note, in both cases at our institution with confirmed central CVF, no central drainage was detected in the upstream DSM. It is conceivable that central draining in lateral DSM could be overlaid by the intrathecal contrast column with single plane x-ray acquisition. Therefore, CTM might be superior to DSM in detection of central CVFs. This could be a subject of future research.

With the “hyperdense basivertebral vein” sign on midsagittal CT, we describe another imaging sign that may help to detect otherwise inconspicuous CVFs.

## Supplementary Information

Below is the link to the electronic supplementary material.

Supplementary file1 (DOCX 983 kb)

## Data Availability

Deidentified information of the two patients can be made available if required.

## Abbreviations

SIH: Spontaneous intracranial hypotension
CSF: Cerebrospinal fluid
CVF: CSF-venous fistula
DSM: Digital substraction myelography
CTM: Computed tomography myelography

## Code availability (software application or custom code)

No custom-made code or commercially available software code was used.

## References

[1] Schievink WI, Moser FG, Maya MM (2014). CSF-venous fistula in spontaneous intracranial hypotension. Neurology.

[2] Kranz PG, Amrhein TJ, Gray L (2017). CSF venous fistulas in spontaneous intracranial hypotension: imaging characteristics on dynamic and CT myelography. AJR Am J of Roentgenol.

[3] Kranz PG, Gray L, Malinzak M (2021). CSF-venous fistulas: anatomy and diagnostic imaging. AJR Am J of Roentgenol in press.

[4] Luetzen N, Dovi-Akue P, Fung C (2021). Spontaneous intracranial hypotension (SIH): diagnostic and therapeutic work-up. Neuroradiology.

[5] Kranz PG, Amrhein TJ, Schievink WI (2016). The "hyperdense paraspinal vein" sign: a marker of CSF-venous fistula. AJNR Am J of Neuroradiol.

[6] Dobrocky T, Grunder L, Breiding PS (2019). Assessing spinal cerebrospinal fluid leaks in spontaneous intracranial hypotension with a scoring system based on brain magnetic resonance imaging findings. JAMA Neurol.

[7] Malinzak SM, Kranz PG, Gray L, et al (2021) Post-surgical recurrence of CSF-venous fistulas in spontaneous intracranial hypotension. Neurology: Clinical Practice Publish Ahead of Print. 10.1212/CPJ.0000000000001061. 10.1212/CPJ.0000000000001061 PMC8382357 34484913

[8] Roytman M, Salama G, Robbins MS, Chazen JL (2021) CSF-venous fistula. Curr Pain Headache Rep 21;25(1):5. 10.1007/s11916-020-00921-4. 10.1007/s11916-020-00921-4 33475890

[9] Brinjikji W, Savastano L, Atkinson J (2021). A novel endovascular therapy for CSF hypotension secondary to CSF-venous fistulas. AJNR Am J Neuroradiol.

