# Supplementary material for: The “hyperdense basivertebral vein” sign: another marker of a CSF-venous fistula
Source: Neuroradiology. 2022 Feb 1;64(3):627–30. doi: 10.1007/s00234-022-02908-x (PMC8850216; doi:10.1007/s00234-022-02908-x)
Supplement: Supplementary file 1 — Supplementary file1 (DOCX 983 kb) [file 234_2022_2908_MOESM1_ESM.docx]

**Supplementary Appendix**

This appendix has been provided by the author to give readers additional information about:

Lützen N. et al. (2021) The “Hyperdense Basivertebral Vein” sign: Another Marker of a CSF-Venous Fistula. Neuroradiology

**Supplementary Appendix**

Supplement to: Lützen N. The “Hyperdense Basivertebral Vein” sign: Another Marker of a CSF-Venous Fistula

**Content**

Supplementary Figure (Fig. 3) + Figure legend page 3

Supplementary Table (Tab. 1) page 4


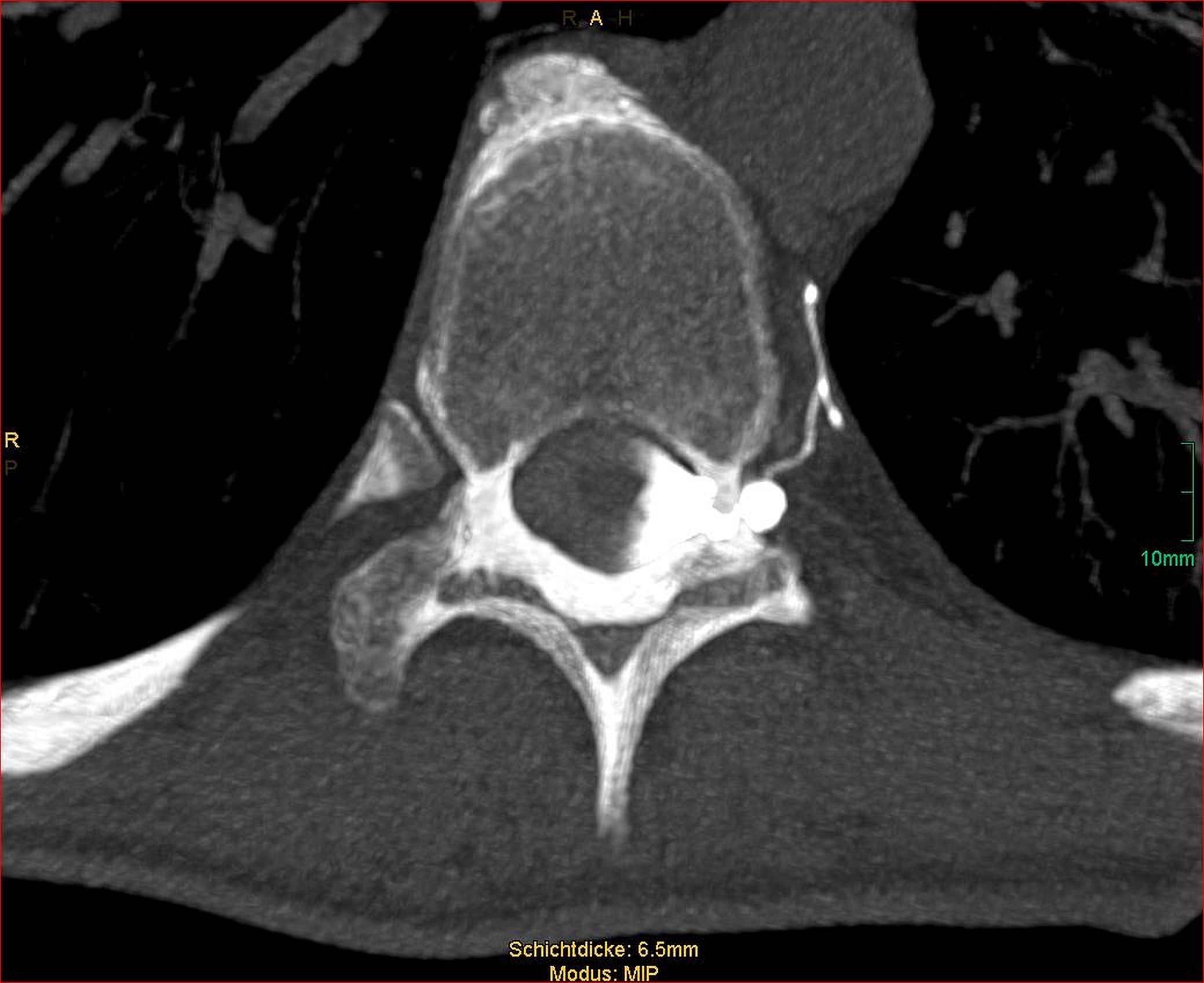


**Fig. 3.** Lateral decubitus CT on the left side after intrathecal injection of iodine contrast, axial MIP reconstruction. This is an example for a well visible paravertebral CSF-venous fistula with a typical CT-appearance in a 56 year old women (not subject of this short report) at the level of Th 10/11 on the left side.

**Table 1: Diagnostic protocol for CSF-venous fistulas at our institution**

1. Digital subtraction myelography (DSM):

- Philips Allura Clarity, Philips Medical Systems, Nederland B.V. or Siemens Artis Icono, Siemens Erlangen Germany
- Lateral decubitus position for lumbar puncture
- Table tilted at 6-7° (head deep)
- Adjustment to the thoracic spine, ap-projection
- Injection of half the dose of contrast (7-8ml), iodine concentration of 300mg/ml
- DSM (1 fps) for a duration of 45 seconds
- Same procedure the next day for the other side

1. CT-myelography (CTM):

- Somatom Definition 64, Siemens Erlangen Germany
- Directly following DSM, with needle in place transfer to the CT scanner in lateral decubitus position
- In lateral decubitus position table (custom made) tilted 6-7° (head deep)
- Injection of remaining iodine contrast (7-8ml)
- Directly following: CT scan from cervical spine to the point of lumbar puncture during inspiration (for example feasible with inspiration through a straw)
- Scan parameters: helical scan mode, rotation time 1.0 seconds, pitch 0.8, reference tube voltage 120 kV, automated exposure control, reference tube current 320 mAs.
- Reconstructions: 0.75mm axial, bone kernel; 3mm sagittal, bone kernel
- Same procedure the next day for the other side
